# Genomic Copy Number Variations Characterize the Prognosis of Both P16-Positive and P16-Negative Oropharyngeal Squamous Cell Carcinoma After Curative Resection

**DOI:** 10.1097/MD.0000000000002187

**Published:** 2015-12-18

**Authors:** Arang Rhie, Weon Seo Park, Moon Kyung Choi, Ji-Hyun Kim, Junsun Ryu, Chang Hwan Ryu, Jong-Il Kim, Yuh-Seog Jung

**Affiliations:** From the Department of Biomedical Science, Department of Biochemistry and Molecular Biology, Genomic Medicine Institute (GMI), Medical Research Center, Seoul National University, Seoul (AR, J-IIK); Department of Pathology, Center for Specific Organs Cancer, Hematologic Malignancy Branch, National Cancer Center, Goyang, Gyeonggi (WSP, MKC); and Department of Otolaryngology—Head and Neck Surgery, Graduate School of Cancer Science and Policy, Specific Organs Cancer Branch, National Cancer Center, Goyang, Gyeonggi, Korea (J-HK, JR, CHR, Y-SJ).

## Abstract

Recently increasing high-risk HPV+ OSCC exhibits unique clinical and molecular characteristics compared to HPV-unrelated (HPV−) counterpart. Genomic copy number variations (CNVs), unique in HPV+ OSCCs, and their role for the prognosis prediction remains poorly studied. Here, we analyzed the distinct genomic copy number variations (CNVs) in human papillomavirus-related (HPV+) oropharyngeal squamous cell carcinoma (OSCC) and their role as a prognosticator after curative resection.

For 58 consecutive, Korean OSCC patients that underwent surgery-based treatment with median 10 years of follow-up, HPV-related markers, and genome-wide CNV analysis were analyzed. Clinical associations between the CNV profile and survival analyses were followed.

p16 expression predicted the overall survival (OS) (hazard ratio [HR] = 0.27, confidence interval [CI]: 0.39–0.80, *P* = 0.0006) better than HPV L1 PCR (HR = 0.83, CI: 0.66–1.29, *P* = 0.64), smoking, or other variables. Although the overall number of CNVs was not significantly different, 30 loci showed unique CNV patterns between the p16^+^ and p16− groups. A region containing PRDM2 was amplified only in the p16^+^ group, whereas EGFR and 11q13.3 showed increased amplification in p16− counterpart. Loss of a locus containing FGF18 led to a worse, but gain of region including CDK10 and RAD18 led to better overall survival (OS) in all OSCC patients. Meanwhile, subgroup analysis of p16^+^ OSCC revealed that amplification of regions harboring HRAS and loss of locus bearing KDR led to better OS.

p16^+^ OSCC exhibit distinct CNV patterns compared with p16− counterpart. Specific patterns of CNVs predict better survival, especially in p16^+^ OSCC. This might allow better insights of the outcome after curative resection for HPV+ and HPV− OSCC.

## Abstract 2

Supplemental Digital Content is available in the text

## INTRODUCTION

Head and neck squamous cell carcinoma (HNSCC) is the eighth most common cause of cancer deaths worldwide.^1^ This cancer can be caused by either human papillomavirus (HPV) or nonviral changes, and each type has its own epidemiologic risks. Interestingly, studies indicate that the incidence of HPV-positive (HPV+) HNSCC has steeply increased in recent years, up to 60% to 85%.^2,3^ HPV+ HNSCC usually occur as oropharyngeal squamous cell carcinomas (OSCC), mostly in tonsillar fossa. They usually respond better to treatments, such as ionizing radiation with or without the addition of chemotherapy^4^ and surgery-based treatments,^4^ than HPV-negative (HPV−) counterpart. This has led some to propose “de-escalating” contemporary treatment strategies through the guidance of molecular markers; for example, decreasing radiation dosage or adopting less invasive, functional surgeries, such as transoral laser microsurgery^5^ and transoral robotic surgery.^6^

In spite of these advances, treatment failures still occur, and the failure rate is increasing due to increased incidence of this disease worldwide. In general, 20% to 40% of HPV+ and 40% to 60% of HPV-HNSCCs still exhibit poor responses to treatment, with patients suffering from disease recurrence.^4,7,8^ Thus, a more detailed understanding of these poor responders is necessary to provide better, functional treatment and improve patient outcomes for this expanding disease.

Copy number variations (CNVs), especially those affecting signaling mediators, might represent a critical factor driving cancer development and determining sensitivity to contemporary anticancer treatments. Losses of 4q, 5q, 7q, 8p, 13q, 17p, 18q, 21q, and 22q and gains of 1q, 2q, 3q26, 5p, 7p, 8q, 9q, 11q13, and 20q have been reported in HNSCCs in Western populations.^9–11^ Among these CNVs, gains of 3q, 11q, and 12q and losses of 5q, 6q, 8p, 21q, and 22q were reported to predict poor prognosis.^9,12^ Also in HNSCC, CNVs of EGFR^13^ and PIK3CA^14^ have also been reported to be associated with poor prognosis. Meanwhile, studies of uterine cervical cancers have provided preliminary knowledge on unique CNV signatures, such as gains of 1q in early stages of tumor development, and gains of 3q, 5p, and 8q and losses of 2q, 3p, 4q, 11q, and 19p later in tumor progression.^15,16^ Integration of HPV DNA into the host genome causes changes like a gain of 3q, which is related to cancer progression in uterine cervical premalignant lesions.^17^ As these studies were mostly on uterine cervical cancers, unique CNV patterns of HPV + HNSCCs, especially compared with HPV− counterpart, should be clarified more. Moreover, most data have been obtained from Western populations, whereas little is known about these patterns in Asian countries such as Korea. Finally, these data were mostly analyzed in a heterogeneous treatment group. There has been little evaluation of the clinical and prognostic implications of CNVs in OSCC, especially after surgical resection as a primary treatment.

Here, we report and elucidate the specific patterns of CNVs in HPV+ and HPV− OSCC in a Korean population. We compared these patterns in p16^+^ and p16− OSCCs. The prognostic role of CNV profile in a consecutive, surgery-treated cohort with OSCC was determined, particularly in the p16^+^ subset, and we evaluated if the CNV pattern provides more accurate stratification for proper treatment.

## MATERIALS AND METHODS

### Subjects and Material

Tissue samples from 58 consecutive, histopathologically confirmed, previously untreated OSCC were obtained during surgical procedures carried out between 2002 and 2007 (50 males, 8 females; age range, 45–82 years; mean age, 61.6 ± 7.4 years), at the Department of Otolaryngology—Head and Neck Surgery, Specific Organs Cancer Branch, National Cancer Center, Goyang, Korea. After obtaining informed consent, archives of paraffin-embedded specimens from these patients were constructed. Inclusion criteria for this study were histologically confirmed primary OSCC with primary surgical resection, with or without postoperative radiotherapy. Exclusion criteria included recurrent disease, contraindication of surgery-based treatment, initial presentation with distant metastasis, failure to receive the full course of planned treatment, or cases where formalin-fixed paraffin-embedded (FFPE) specimens lacked sufficient DNA for CNV analysis.

Clinical data, including tumor staging and details of primary treatment, were obtained from medical, radiology, operative, and pathology reports using the 2002 American Joint Committee on Cancer staging criteria.^18^ Histologic grading was performed by a pathologist according to the World Health Organization criteria for squamous cell carcinomas of the oral mucosa.^19^ All OSCC patients were examined by the head and neck surgeon at each follow-up appointment (every 3 months). Cumulative tobacco and alcohol use were calculated as previously described.^8^ This study was approved by the Institutional Review Board in accordance with the Center for Clinical Trials at the National Cancer Center, Korea (NCCNCS08200).

### Treatments

All patients underwent surgery-based treatment, mostly with transoral lateral oropharyngectomy through en-bloc dissection of the submuscular plane along the buccopharyngeal to the prevertebral fascia, as previously described.^20^ Oropharyngectomy, via a more aggressive transpharyngeal or transmandibular approach, was also performed if indicated. Neck dissection was routinely accompanied, either with elective (cN0) or therapeutic (cN+) intent. Adjuvant radiotherapy was done in patients with positive or close (<2 mm) surgical margins, demonstrated perineural or lymphovascular invasion, any stage N2 neck disease, or neck disease with extracapsular extension after tumor board discussion on oncologic necessity.

### Immunostaining and Scoring

**FIGURE 1:**
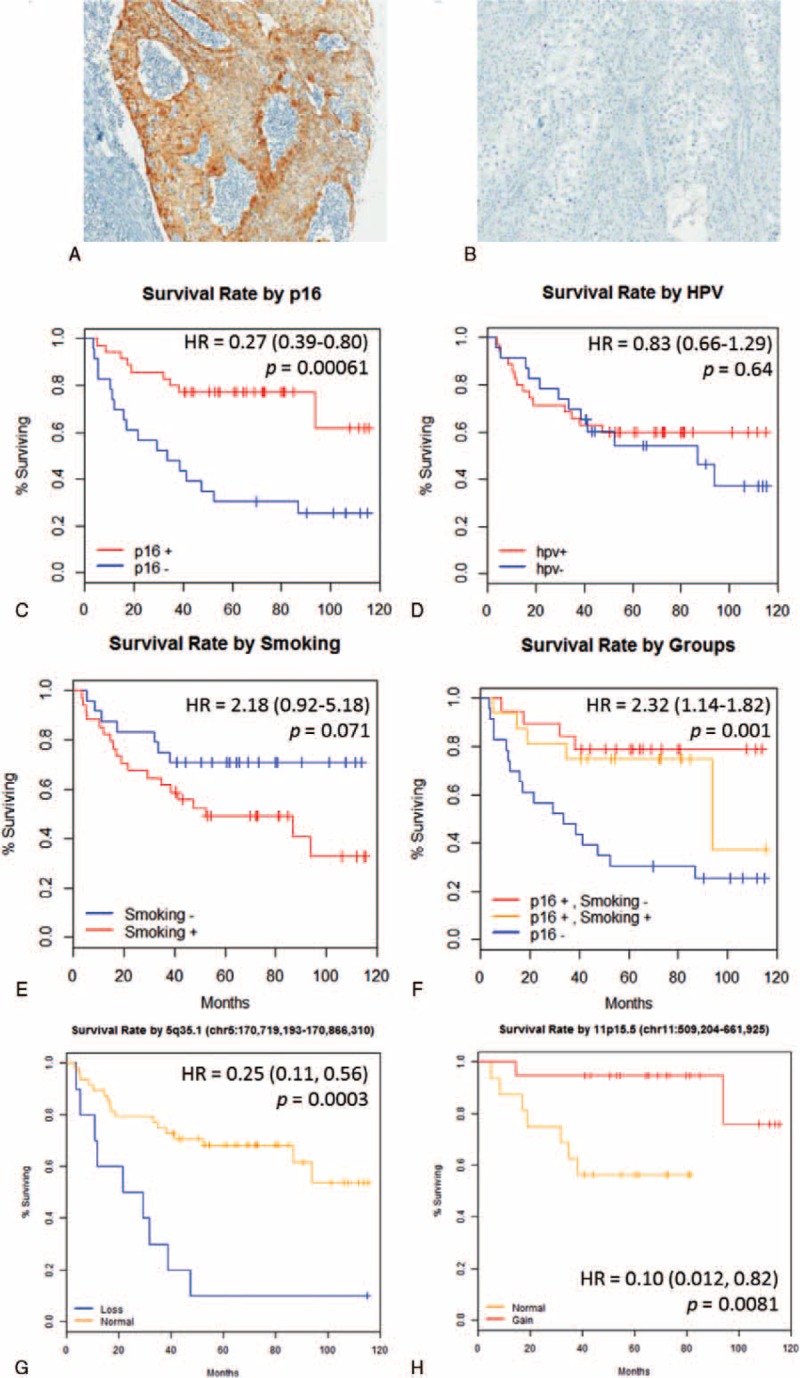
Representative images of the typical strong (A) and negative (B) expression of p16 following immunohistochemical analysis of OSCC tumor specimens: original magnification, 100×; Kaplan–Meier overall survival by (C) p16 expression, (D) HPV-L1 DNA PCR, and (E–F) smoking history, for initial characterization for further stratification. This basic stratification showed that p16 predicts the overall survival most significantly. (G) Survival curve for copy number variation of 5q35.1 (FGF18) in all enrolled patients (n = 58), and (H) of 11p15.5 (harboring *HRAS*) in p16^+^ oropharyngeal squamous cell carcinoma (n = 35), showing a significant prognostic difference. Hazard ratio from Cox proportional hazard regression with 95% confidence interval. *P* values have been obtained from the log-rank test. HPV = human papillomavirus; HR = Hazard ratio; DNA = deoxyribonucleic acid; PCR = polymerase chain reaction; OSCC = oropharyngeal squamous cell carcinoma.

**FIGURE 2:**
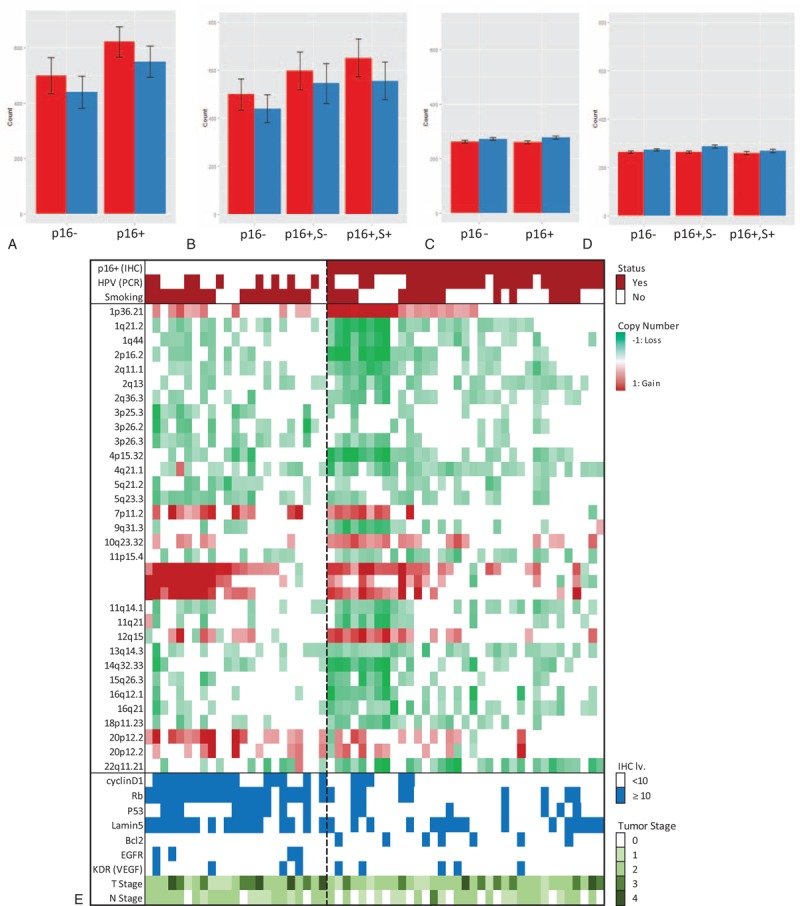
Gross pattern of copy number variation (CNV) between p16^+^ (smoking+/−) versus p16− oropharyngeal squamous cell carcinomas. We defined probes with a log2ratio > 0.30 as “gain”, < −0.30 as “loss”, and between −0.30 and 0.30 as “normal.” (A and B) Mean total number of copy number gain (red bars) and loss (blue bars). (C–D) Mean total number of consecutive (long CNV as >5 consecutive probes) CNV. Student's 2-tailed *t* test was used to obtain statistical *P* values for average copy number differences between p16− and p16^+^ groups. Analysis of variance (ANOVA) has been applied to analyze group means difference within groups of p16−, p16^+^ with and without smoking history. On average, copy numbers were not significantly different between groups in overall. (E) Heatmap of chromosomal copy number alterations that are significantly different between p16− and p16^+^ groups. Detailed *P* values and genes locating in the region are stated in Table 2. 1q36.21 is highly amplified in p16^+^ groups, and 11q13.3 is more amplified in p16− groups. Copy losses are more frequently observed in the p16^+^ group. Different patterns of CNVs are suggesting altered pathways in tumorigenesis. Additional immunohistochemistry results are provided below the heatmap. S, Smoking. IHC lv, immunohistochemistry composite score (highest possible score: 30). ANOVA = analysis of variance; CNV = copy number variation; S = smoking.

A tissue microarray was constructed.^21^ Immunohistochemistry (IHC) of p16 p16 (1:50; P2D11F11; Novocastra, Buffalo Grove, IL) was performed and scored using modified H-score system, which considers both the intensity and the proportion as follows:^8,22,23^ (1) the staining intensity was defined as 0 for negative, 1+ for weak, 2+ for moderate, and 3+ for strong (Fig. 1A and B); (2) the positive area was defined as the (10×) fraction of stained tumor cells in the entire tumor; (3) expression score was defined as the staining intensity multiplied by the percent of the positive staining area. The highest possible score was 30. A high expression of each marker was defined by a score >20. An experienced head and neck pathologist (W.S.P.), who was blind to the clinical information, comprehensively reviewed and scored the expression profile.

Immunostaining and statistical analysis were performed also at the stage of validation. For choosing candidates for validation, we prioritized genes recurrently showing amplification/deletion pattern in The Cancer Genome Atlas (TCGA) dataset among the genes passing the survival test. Then, immunostaining was conducted for the same sample set. Correlation between the CNV pattern and the IHC expression was further tested with Fisher's exact test, followed by survival analysis using the IHC expression.

### DNA Extraction

DNA was extracted using the QIAamp Mag Attract DNA Mini M48 Kit (Qiagen, Valencia, CA). The carcinoma area was punched out from paraffin blocks to obtain the highest percentage of tumors and collected in 1.5-mL Eppendorf tubes for DNA extraction. DNA extraction was carried out using the Qiagen BioRobot M48 workstation. A total of 10 μL of purified total cellular DNA was used in each HPV PCR.

### HPV Genotyping in an HPV Chip

The presence of HPV DNA was tested simultaneously with genotyping using a PCR-based HPV DNA Chip (Greencross, Gyeonggi, Korea), as previously described.^8^ Fifteen types of high-risk HPV (HPV-16, 18, 31, 33, 35, 39, 45, 51, 52, 53, 56, 58, 59, 66, 68) and 9 types of low-risk HPV (HPV-6, 11, 34, 40, 42, 43, 44, 54, 70) were identifiable with this chip.

### Array Comparative Genomic Hybridization

The array used in this study (MacArray Karyo, Macrogen, Korea, http://www.macrogen.com) consisted of 4362 human bacterial artificial chromosome (BAC) clones spaced ∼1 Mb across the entire genome. Confirmation of the locus specificity of the chosen clones was performed by fluorescent in situ hybridization (FISH),^24^ and the labeling and hybridization protocols were used as previously described.^25^ Test and reference DNA were digested, purified, and labeled by random priming (BioPrime-Array CGH Genomic Labeling System; Invitrogen, Carlsbad, CA) using Cy3 or Cy5 dCTPs (GeneChem Inc.; Daejeon, Korea). A Cy3-labeled sample and Cy5-labeled reference DNA were then hybridized, and the arrays were scanned into 2 16-bit TIFF image files and quantitated using the GenePix software (Molecular Devices, Sunnyvale, CA).

### Data Processing and Statistical Analysis

A total of 4362 different BAC clones were used, and the log-transformed fluorescent ratios were calculated. Probes in the autosome region were selected for this study. For quality control, probes genotyped with >90% of the samples were used excluding singleton peaks. We defined probes with a log_2_ ratio > 0.30 as “gain,” < −0.30 as “loss,” and between −0.30 and 0.30 as “normal.” Moreover, regions spanning > 5 consecutive probes at close genomic locations ( < 1000 bp) were defined as consecutive CNVs. To test differences between groups in survival curves, we used Harrington and Fleming's G-rho family test (*survdiff*) from the R package (www.r-project.org) *survival*. Statistical *P* values were obtained with a chi square test. Student's *t* test was used to compare the overall copy number changes between groups, as well as to rank genetic loci. We also performed average-linkage hierarchical clustering based on the centered correlation measure (Cluster 3.0). To determine how genes affect survival rate, copy number gains, and losses were counted. Among probes with copy number gain or loss in >10 patients, survival analysis was conducted using the Kaplan–Meier (*survfit*) method and the proportional hazard regression model (*coxph*). Significance between differences in the survival rate was analyzed by the log-rank test. Results with *P* values <0.05 were considered significant.

## RESULTS

We first analyzed the prognostic significance of principal candidate prognosticators, such as HPV L1 PCR, p16, smoking, and other clinico-pathologic variables. The median follow-up period was 10.69 years (range, 8.2–13.31 years). Among these, the expression of p16 by IHC had the lowest hazard ratio (HR) (0.27, 95% confidence interval [CI] 0.39–0.80, *P* = 0.0006) and more significantly predicted the 10-year overall survival (OS) than HPV L1 PCR (HR = 0.83, CI 0.66−1.29, *P* = 0.64) or smoking (HR = 2.18, CI 0.92–5.18, *P* = 0.071) (Fig. 1C–E). Although smoking has been reported as an effective prognosticator, especially within the p16^+^ OSCC group, our data revealed that smoking is not a significant prognosis factor, and even within p16^+^ OSCC (HR = 1.60, CI 0.43–5.95, *P* = 0.48, see Supplemental Figure 1). Both p16^+^ smokers and p16^+^ nonsmokers responded better to treatment than p16− OSCC (HR = 1.73, CI 1.24–2.41, *P* = 0.00057, Fig. 1F). Other clinical variables, such as demographics, TNM stage, tumor margin, extranodal spread, and treatment details, did not impact OS by univariate analysis (see Supplemental Table 1). Hence, we decided to perform further analyses focusing on p16 expression, initially concluding that this would be the most distinct molecular marker of our cohort. Further analyses of basic clinic-demographic parameters revealed no differences between p16^+^ and p16− OSCC in terms of sex, amount of smoking, or TN stage. Furthermore, the details of surgical resection, resection margin status, or the proportion of the cases that had postoperative radiotherapy were similar between the 2 groups. The 2 parameters that showed differences were age and subsite of OSCC occurrence. p16^+^ OSCC occurred more frequently in patients >60 (*P *= 0.049) and in the tonsillar fossa area (*P* = 0.004, Table 1).

**Table 1:**
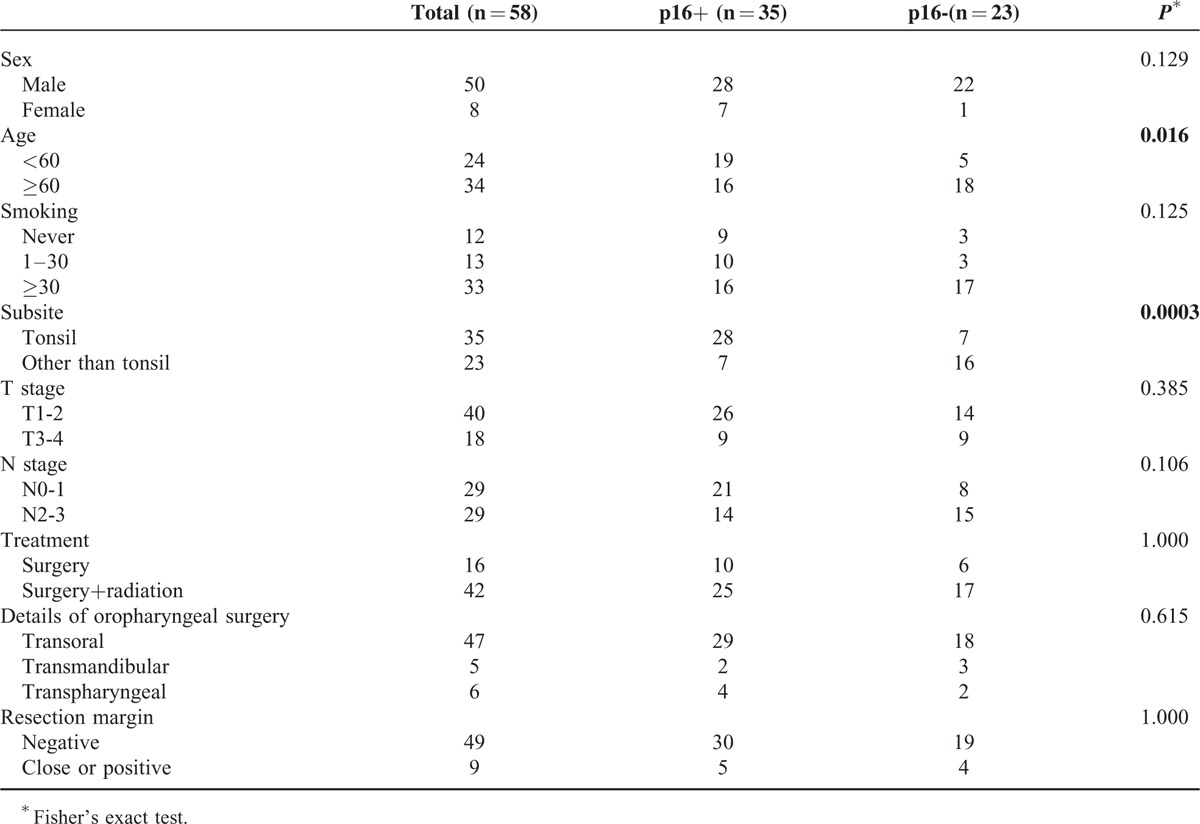
Baseline Patient Characteristics

Although we initially hypothesized that p16^+^ OSCC might have fewer CNVs and milder chromosomal aberrations, the mean number of gain, loss, or their sum were similar between the p16^+^ and p16− groups (*P* > 0.05; Fig. 2A and B). Copy number variations (CNVs) detectable on consecutive probes were also similar between the groups (Fig. 2C and D). We then compared the parameters of general genomic changes between p16^+^ smokers, p16^+^ nonsmokers, and the p16− group. However, no differences were observed between the groups (*P* > 0.05; Fig. 2A–D) on a gross scale. Similarly, as shown in the heatmap, unsupervised clustering did not differentiate the p16^+^ and p16− groups (see Supplemental Figures 2 and 3).

We nevertheless attempted to compare CNVs on autosomal chromosomes in relation to the p16 expression. Notably, a gain in the copy number of EGFR was more strongly observed in the p16− group. Greater gene amplification was observed in p16− OSCC than p16^+^ OSCC at 11q13.3, which contains CTTN, PPFIA1, and SHANK2. Close to this region, CCND1 was also amplified. In contrast, various loci at 3p had copy number losses or chromosomal deletions in p16− OSCC. CNTN4, some micro RNAs, and other regulatory elements are located in this area (regulatory elements are not shown). In contrast, 1p36.21, a locus containing PRDM2, was amplified only in the p16^+^ group. Losses in other chromosomal locations were observed only in the p16− group (Table 2).

**Table 2:**
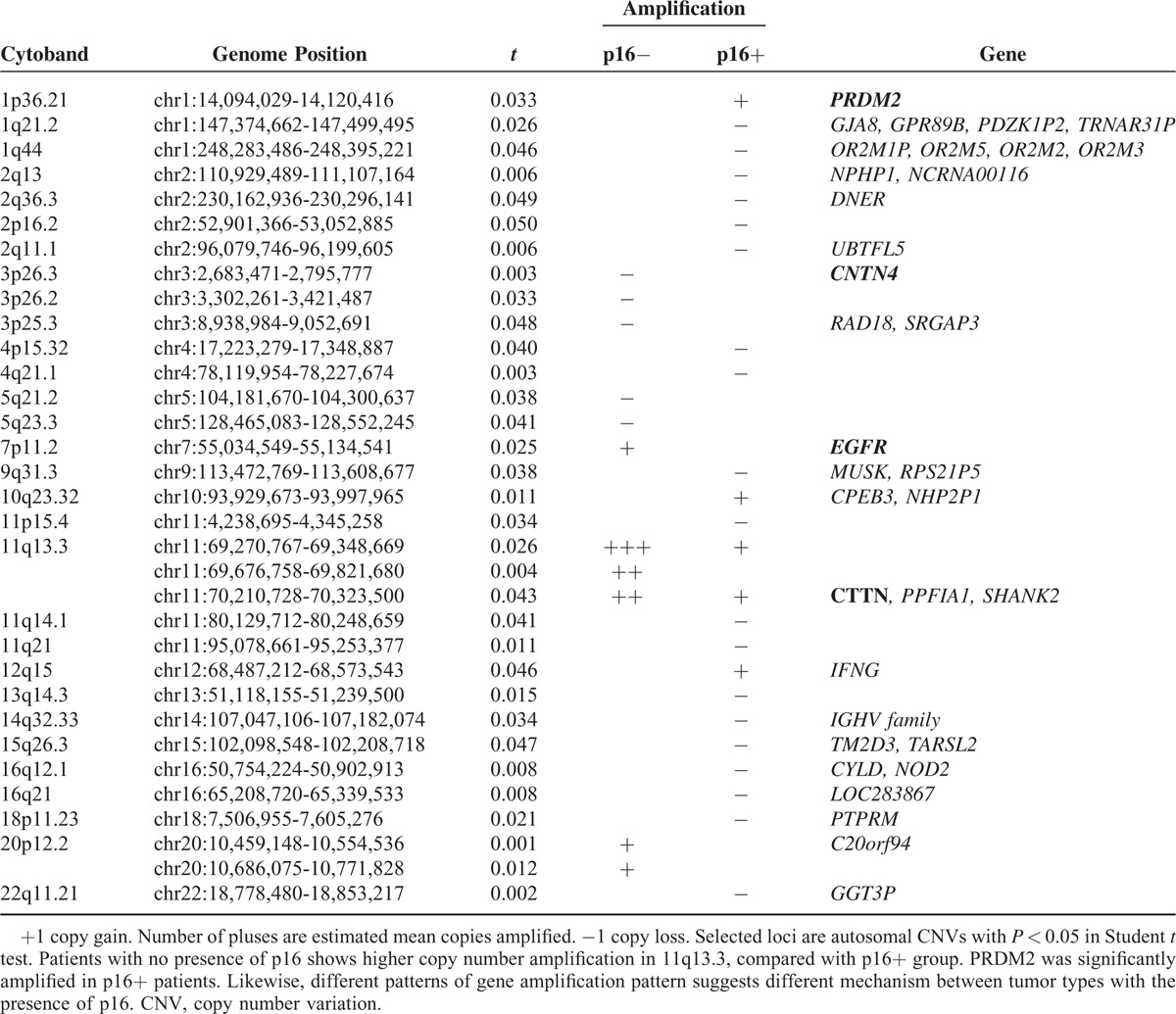
Difference of Pattern of CNVs by p16 Immunohistochemistry Status, Listed in Chromosomal Order Between p16− and p16^+^ OSCC Patients, and Grouped When Cytoband Overlaps

We eventually compared the prognostic impact and significance of CNV patterns to overall survival in all surgically treated OSCC cohorts enrolled. Copy number loss of 5q35.1, harboring genes such as FGF18 (Fig. 1G) and 17p12 predicted a poorer OS outcome, whereas loss of 16q24.3 encoding genes such as CDK10, and 3p25.3, encoding genes such as RAD18, predicted a better OS. A copy number loss of 20p12.1, encoding PCSK2, 7q31.2, and 4q21.21, significantly predicted a better OS (see Table 3, Supplemental Table 2 and Supplemental Figure 4).

**Table 3:**
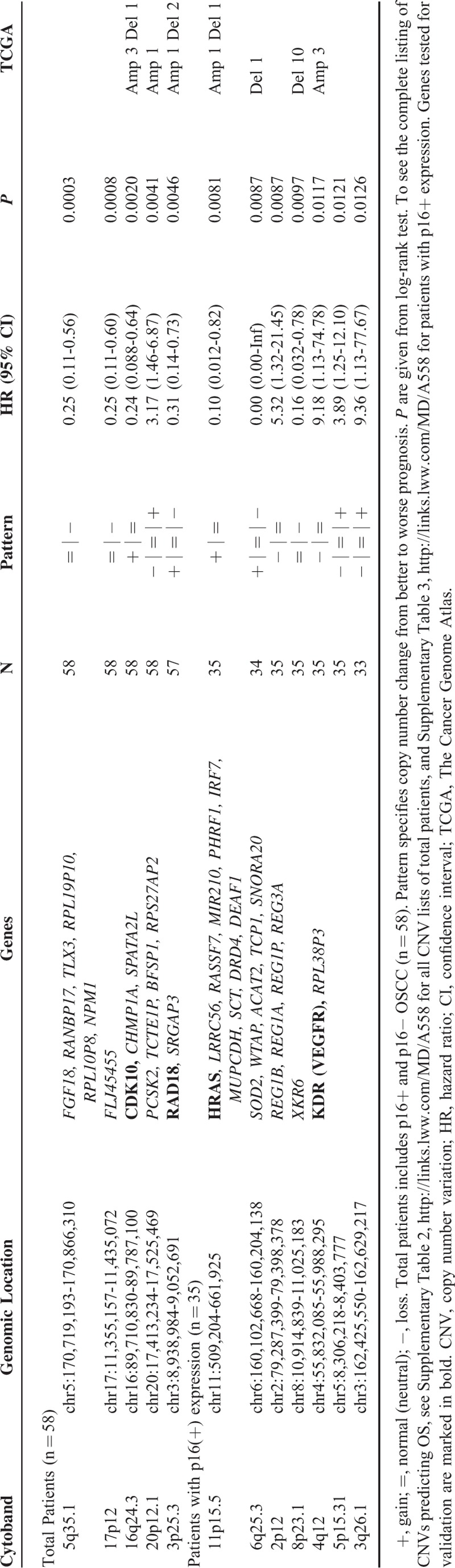
Top 5 CNV Predicting Overall Survival Most Significantly, Seen With Cox Proportional Hazard Model

As an effort to identify better prognosticators within p16^+^ OSCC after surgical resection, we performed further subgroup analysis within this population. In total, 44 CNV patterns showed significantly different predictions of OS (Table 3, Supplemental Table 3). Some patterns with a higher copy number of autosomal loci predicted better survival, such as 11p15.5, encoding several genes including HRAS (Fig. 1H). On the other hand, a copy number loss of 4q12, bearing genes such as KDR, predicted a better OS. Survival curves for these CNVs are presented in Supplemental Figure 5.

## DISCUSSION

In this study, the subjects were 58 consecutive Korean patients, who had oropharyngeal cancer without prior treatment. All subjects underwent homogenous surgical resection, with or without radiation therapy. We herein analyzed median 10-year follow-up data after initial treatment, to elucidate whether chromosomal changes could predict the prognosis after surgery-based treatment for OSCC. Little has been known about the role of genomic CNVs as prognostic markers, especially in HPV+ OSCC, which have been increasing recently in many countries.

Our initial analysis on primary parameters indicated that p16 was the most potent prognostic marker (*P *= 0.00391) compared with other markers, such as HPV- L1 PCR (*P *= 0.0263) and smoking (*P *= 0.38). We thus continued to search for post-treatment prognosticators, focusing on p16 expression, speculating that this might stratify our cohort with more clinical and biologic relevance. Despite recent reports suggesting that a combination of HPV DNA and p16 positivity is the most reliable indicator of an active HPV association,^26^ our data indicated that p16 positivity alone could better predict the OS than HPV L1-PCR. This is in line with some previous reports.^27^ Although DNA analysis in FFPE is a well-established standard, genomic DNA in an FFPE specimen can become fragmented over time, decreasing the accuracy of HPV L1 PCR. We think that this could lower the accuracy of HPV DNA testing in archival FFPE samples than p16 IHC. In addition, although smoking has occasionally been reported as a prognostic marker for OSCC, smoking history did not predict survival in p16^+^ patients, in agreement with another previous report.^28^ It is still unclear whether there is racial disparity in the influence of smoking, which is not well understood in the Asian population.

Although a few reports from the United States and European countries have described a significantly lower total number of chromosomal alterations per tumor in the HPV+ OSCC compared with HPV− OSCC,^29,30^ the general landscape of our cohort was different. The total number of CNV gains and losses were not different in a statistically significant manner between p16^+^ and p16− patients in our cohort. Unsupervised clustering also failed to group patients by p16 status. Large losses or gains in consecutive CNVs were not different between p16− smokers, p16^+^ nonsmokers, and p16− OSCC (*P* > 0.05). However, the loci of CNVs showed different patterns (see Fig. 2E, Table 2 and Supplemental Figure 3).

Among chromosomal locations showing the most CNV differences between p16^+^ and p16− OSCC, 11q13 ranked highest, followed by 22q11.21, 1q36.21, and 7q11.2. This overall amplification pattern is in line with recent TCGA data.^31^ The amplification pattern was shown particularly on 11q13.3 among p16− patients, implying different carcinogenetic mechanisms between OSCC subgroups (see Fig. 2E, Table 2, and Supplemental Figure 3).

The viral protein E6 promotes the degradation of p53 and E7 inactivates pRb, usually followed by viral integration into the host genome.^32,33^ Recently, a genome-wide study using whole genome sequencing revealed HPV integration sites associates with recurrent focal genomic instability.^34^ This study showed a small fraction of HPV 16 directly integrated into the midst of a chromosomal translocation, juxtaposing between 11q13 and 8p11, implying that CNV in HPV+ cancers might exhibit unique patterns, connected to the viral integration process.

CCND1, which is located close to 11q13, encodes Cyclin D1 and is frequently detected by IHC on samples from p16− patients (69.6%) compared to p16^+^ patients (14.2%). Cyclin D1 is known to be overexpressed with inactivated p53 in immortalized cells.^35^^.^^36^ TP53 loss or deletion was present in 70% of our current patients, regardless of the p16/infection status (73% of p16− and 68% of p16^+^ patients, Fig. 2E). Gene amplification of EGFR may also affect cell cycle progression by induction of Cyclin D1 and its downstream oncogenic growth factor-signaling pathway. Notably, EGFR is less amplified in p16^+^ OSCCs compared to p16−. This result may explain the low frequency of EGFR amplification in HNSCC from other studies, generally reported as 10% to 30%.^37,38^ Patients showing EFGR amplification might represent a better candidate for EGFR-targeted chemotherapy.

Together with CCND1, CTTN is reported to correlate with prognostic tumor stage and contribute to metastasis in HNSCC, often coinciding with resistance to anoikis.^39–41^ PFFIA1 in 11q13 is also reported in several earlier studies.^42,43^ We also found a copy number gain in 1p36.21. We suspect one of the genes in this locus, PRDM2 (also known as RIZ), to be involved, as the role is known as a tumor suppressor. Another study on expression patterns based on the p16 status revealed PRDM2 overexpression in p16^+^ patients, consistent with our result.^44^

We eventually investigated the role of cancer-specific CNVs as prognostic markers in surgically treated OSCCs, especially the p16^+^ subset, which has been rarely studied especially after surgical resection. Although EGFR,^45^ CCND1,^39^ MYC, FGFR1,^46,47^ and PIK3CA amplification have been reported by a number of studies on HNSCC, their role as a prognostic marker with or without the presence of p16 infection is unclear. Among patients in this study, neither of them showed a significant difference in survival: not in all patients, p16^+^, nor in p16− subgroups (see Supplemental Table 4, Supplemental Figures 6 and 7). Instead, other CNVs have been suggested to be correlated with better or worse survival. In both p16^+^ and p16− subsets, higher copy numbers of 5q35.1, a region harboring FGF18, 16q24.3, containing CDK10, and 3p25.3, containing RAD18, typically predicted a better OS. Although the roles of FGF18 and CDK10 in cell cycle progression and proliferation have been well studied, the relationship between their copy number and cancer survival is not fully understood. CDK10 has been reported as a tumor suppressor gene in biliary tract cancer cells.^48^ RAD18 is involved in telomere maintenance, concordantly showing better survival with copy number gain. Conversely, a higher copy number of 20p12.1, encoding genes such as PCSK2, predicted a poorer OS in our group. PCSK2 is a factor related to tumor development and progression. Recently reported expression analysis and IHC classified PCSK2 as 1 of 3 genetic markers for the classification of benign and malignant thyroid cancer.^49^

To better predict the OS within our p16^+^ subgroup, we further analyzed the CNV profile. Of note, a higher copy number of 11p15.5, containing HRAS, predicted a better survival outcome. Although HRAS is a known oncogene, it has been reported that activation of HRAS is not sufficient to maintain a neoplastic phenotype.^50^ This suggests another mechanism for the involvement of HRAS in cancer prognosis and survival. Loss of KDR (also known as VEGF receptor) predicted a better prognosis among our p16^+^ patients. Because an increase of KDR promotes angiogenesis, loss of KDR leads to a better OS. Of note, we think these patterns could help guide a “de-escalation” strategy for p16^+^ OSCC treatment.

Meanwhile, the IHC results of genes RAD18, HRAS, KDR did not significantly correlate to the CNV profile and failed to show significance to overall survival (data not shown). Although not significant, HRAS in the p16^+^ group showed a tendency to be correlated between CNV and IHC tests (*P* = 0.07, Fisher's exact test), and the survival of low expression of IHC patients was better than its counterpart (Supplemental Figure 8), although not significant due to the small number of samples in the IHC low group (n = 5). An extended study might be needed.

Our data show that treatment outcome after surgery for OSCC could be more accurately characterized, with the adoption of this array CGH system, and this might help eventually guide optimal treatment. Moreover, our result implicate that less-invasive functional, “de-escalation” surgery would be better performed under stringent guidance from these better prognostic CNV profiles. In addition, considering our data were acquired using “low-resolution” array, reanalyzing the same or independent patient group using the higher resolution array system and further resultant validation would be another enormously important study to be followed soon.

In conclusion, p16^+^ OSCCs exhibit specific CNV patterns at some loci compared with p16− OSCCs, suggesting distinct carcinogenic events at the gene level. Moreover, after surgery-based treatment for this disease, CNV profiles might help more accurately predict treatment outcome after surgery for OSCC and eventually guide optimal treatment strategy. These implicate that less-invasive functional surgery would be better performed under stringent guidance from these better prognostic CNV profiles. Our data also suggest that more intensive treatment and surgical strategy may be needed for OSCC with nonfavorable CNV profile, necessitating further clinical investigations.

## Supplementary Material

Supplemental Digital Content
