# Supplementary material for: Genomic Copy Number Variations Characterize the Prognosis of Both P16-Positive and P16-Negative Oropharyngeal Squamous Cell Carcinoma After Curative Resection
Source: Medicine (Baltimore). 2015 Dec 18;94(50):e2187. doi: 10.1097/MD.0000000000002187 (PMC5058900; doi:10.1097/MD.0000000000002187)
Supplement: Supplemental Digital Content [file medi-94-e2187-s001.pdf]

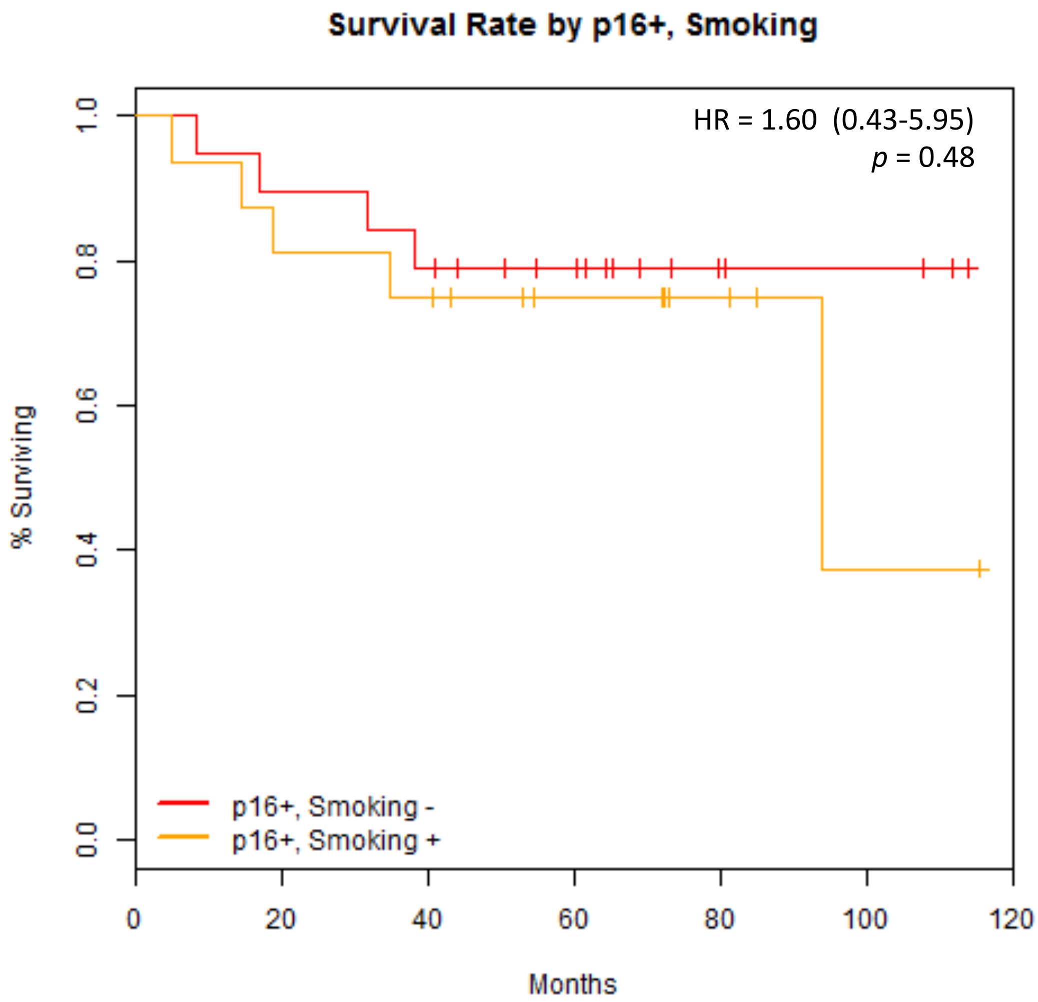

Supplemental Figure 1. Kaplan-Meyer overall survival by smoking history within p16+ oropharyngeal squamous cell carcinoma patients.

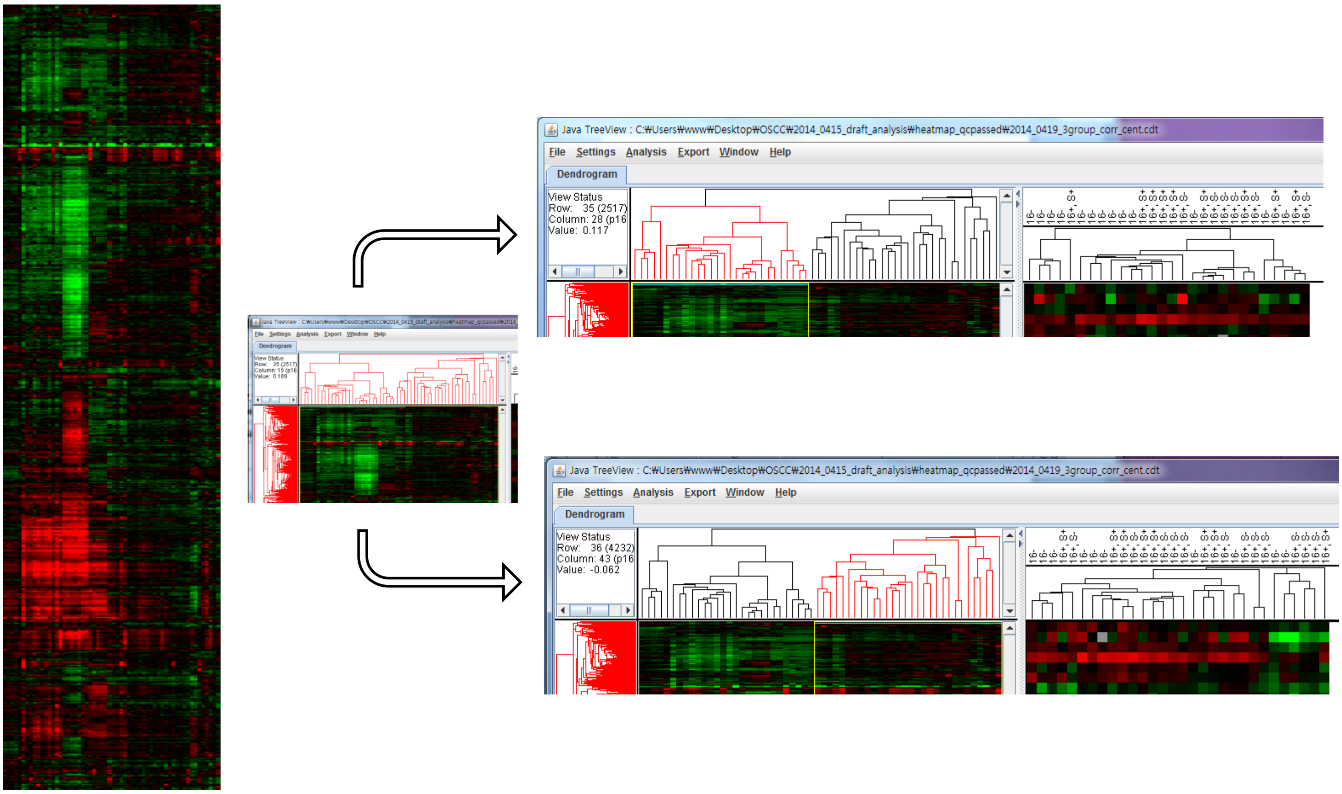

Supplemental Figure 2. Hierarchical unsupervised clustering. Patients were clustered into 2 groups, but hardly clustered with p16 expression or smoking history. S, smoking.

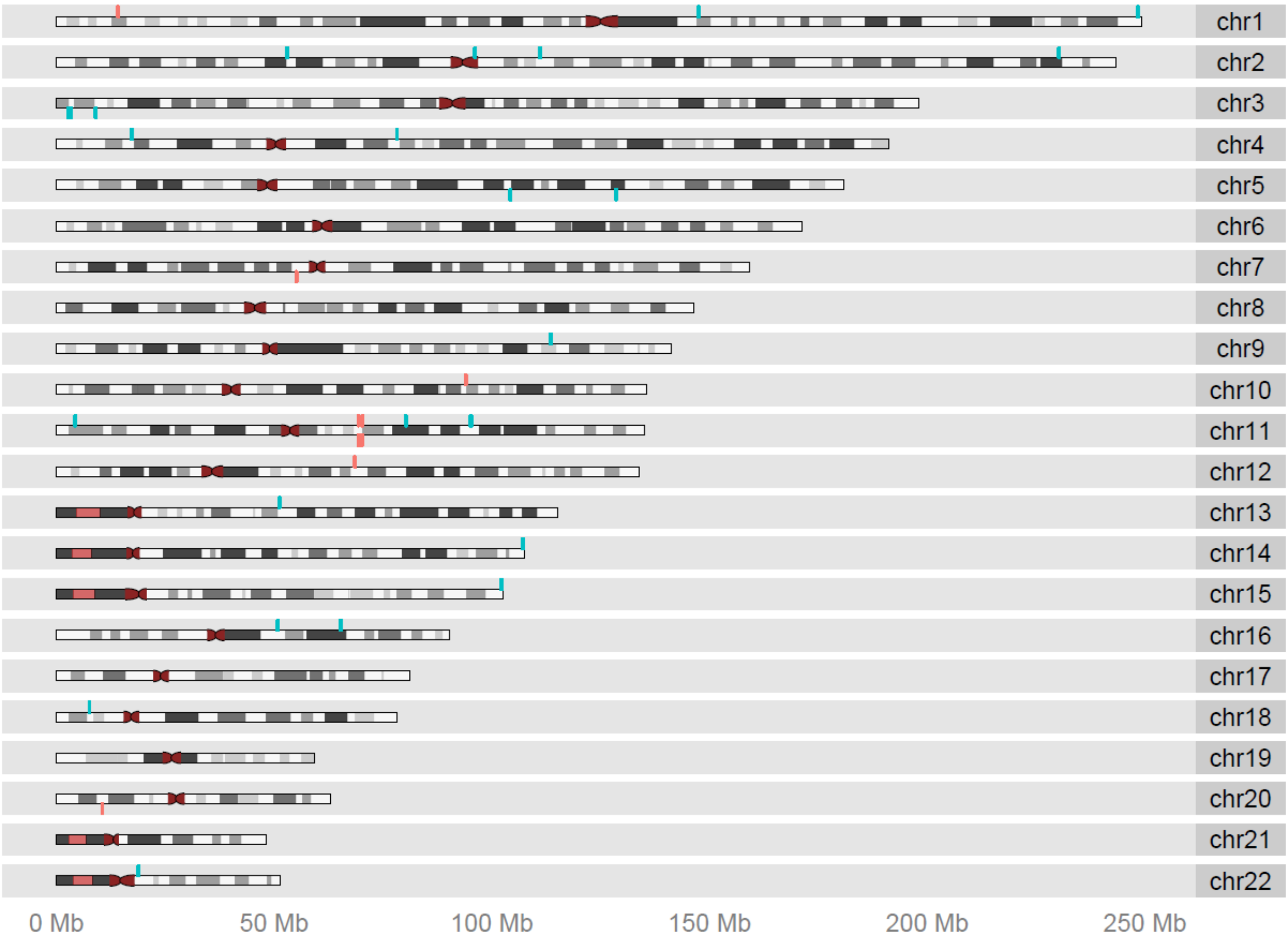

Supplemental Figure 3. Ideogram of copy number variation (CNV) pattern on autosomes that significantly differs between p16+ and p16- groups. Upper bars from the chromosome indicate CNVs in p16+, and lower bars p16-. In these bars, copy number gain are colored in red, whereas losses are in light blue. Copy number gain are observed in 11q.13 in both groups, and copy number losses are more frequently observed genome-widely. Different locations of the copy number alterations are suggesting selective amplification between p16+ and p16- group. Giemsa staining colors were drawn with R package biovizBase hg19.

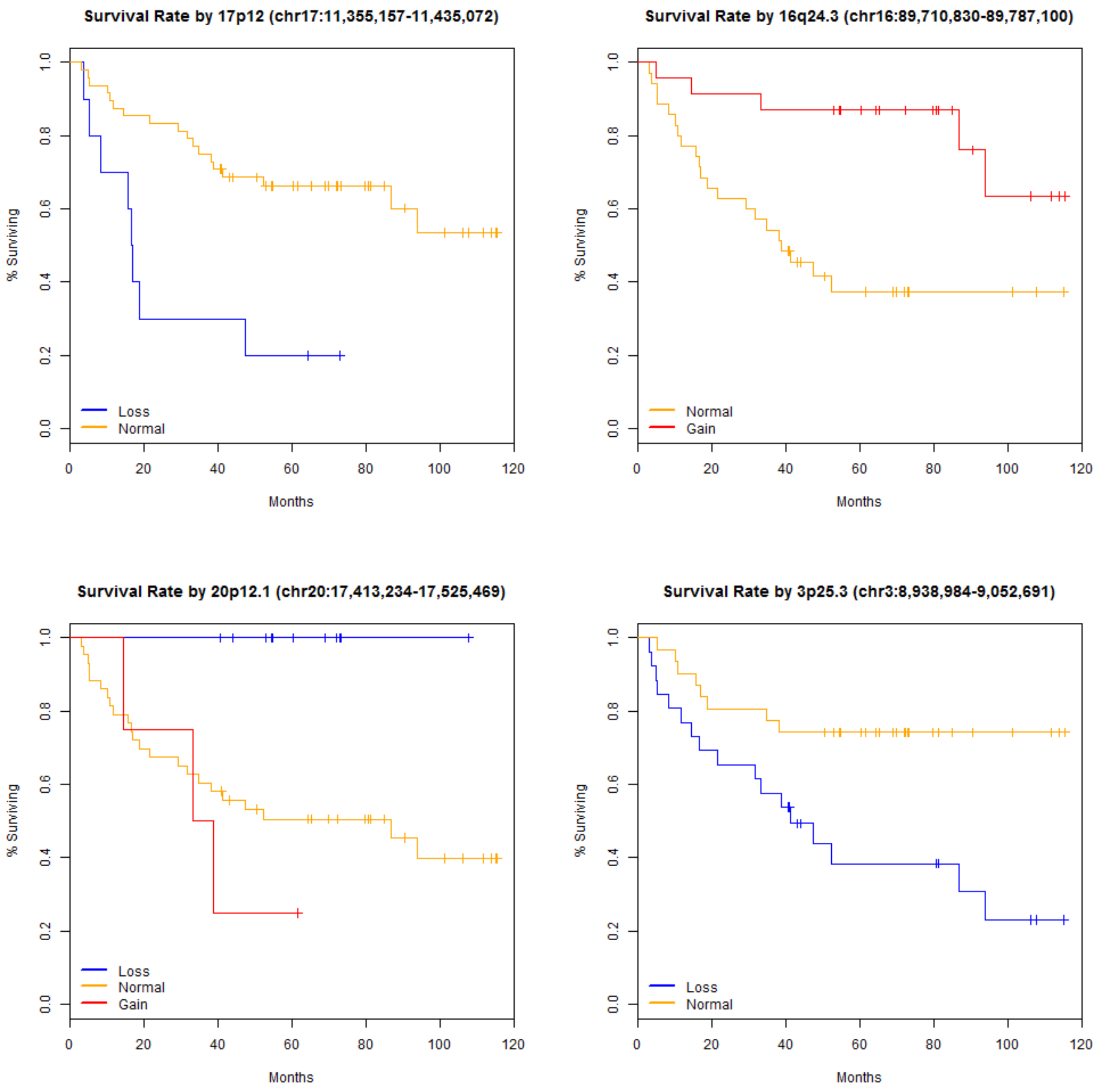

Supplemental Figure 4. Survival curve of copy number variations predicting overall survival, in all patients (n=58). For 3p25.3, 1 gain is included in prognosis pattern of Table 3, but not plotted in this figure.

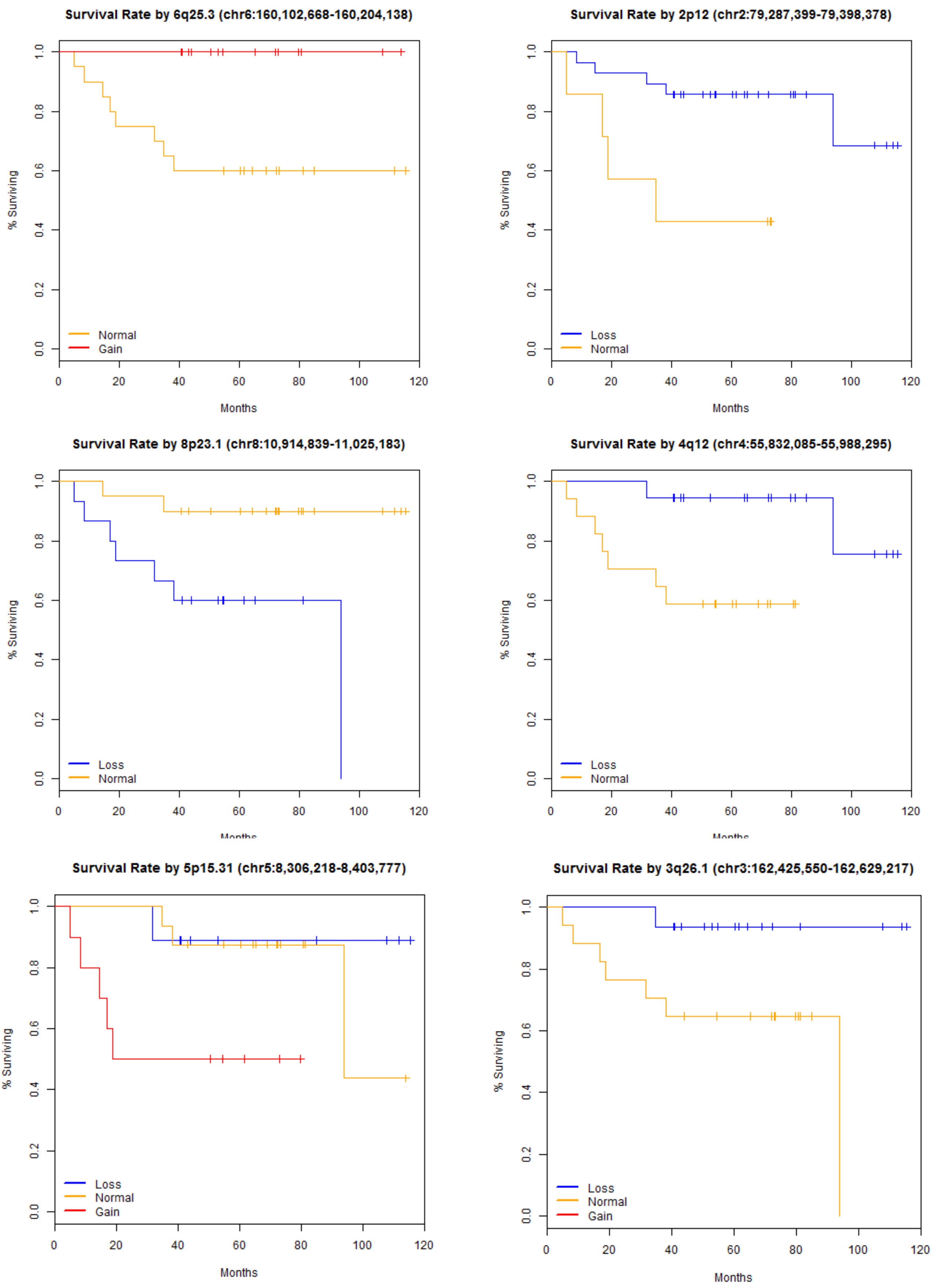

Supplemental Figure 5. Survival curve of copy number variations predic ting overall survival, in p16+ subgroup (n=35). For 6q25.3, 1 loss is includ ed in prognosis pattern of Table 3, but not plotted in this figure. This is al so same for 1 gain at 3q26.1, which is not plotted in this figure.

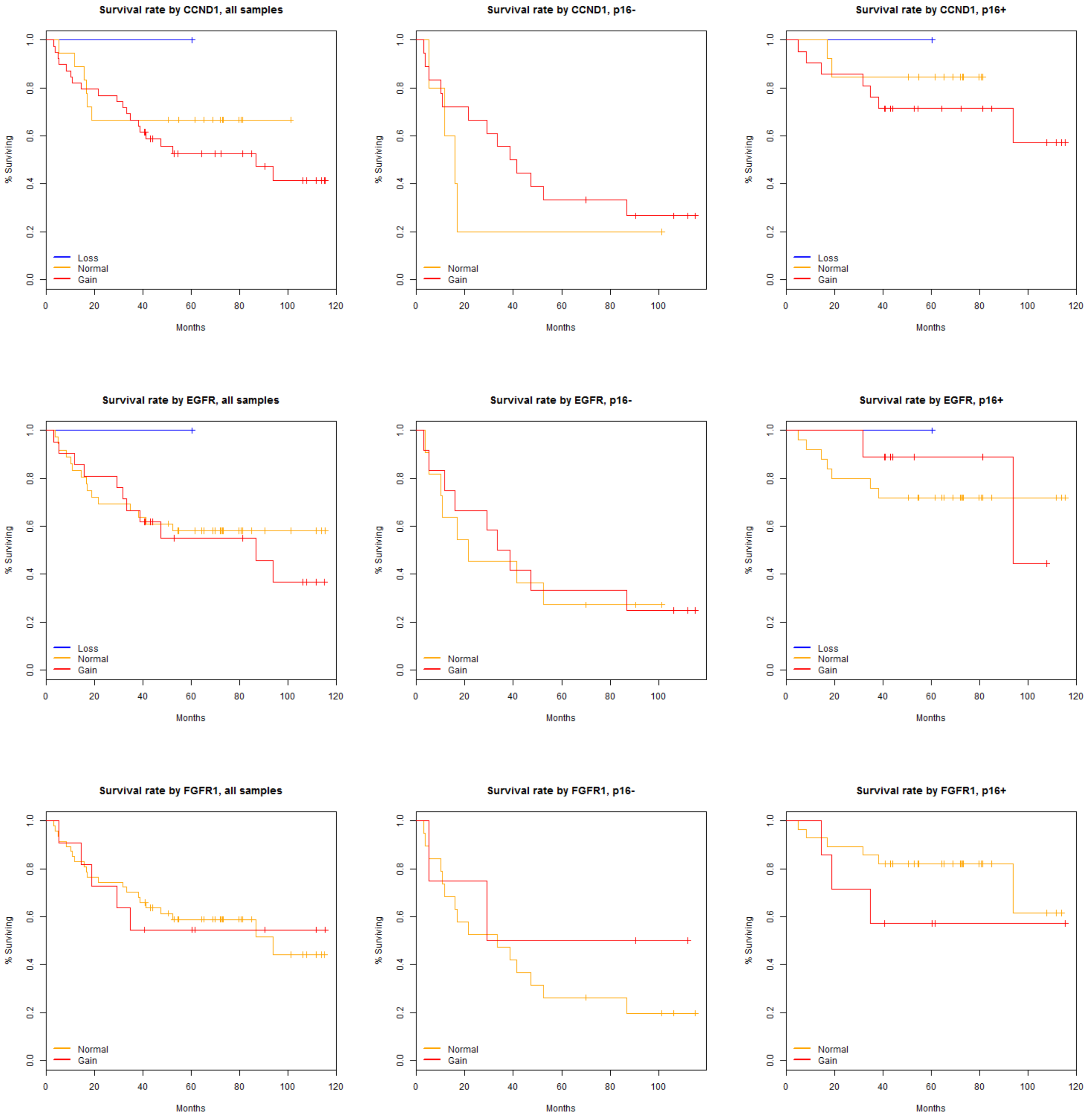

Supplemental Figure 6. Survival curve of copy number variations predic ting overall survival, for genes CCND1, EGFR, and FGFR1.

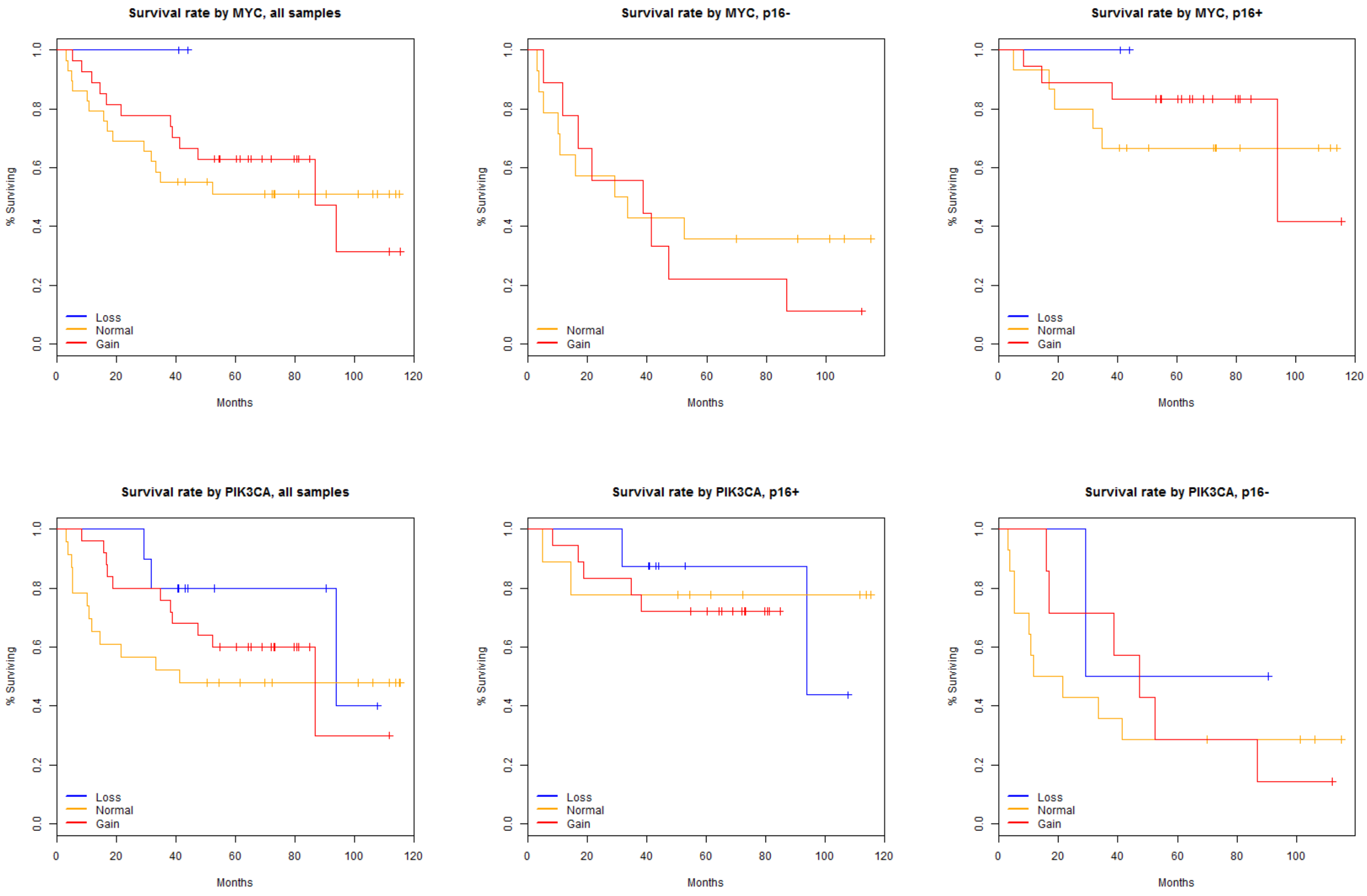

Supplemental Figure 7. Survival curve of copy number variations predic ting overall survival, for genes MYC and PIK3CA.

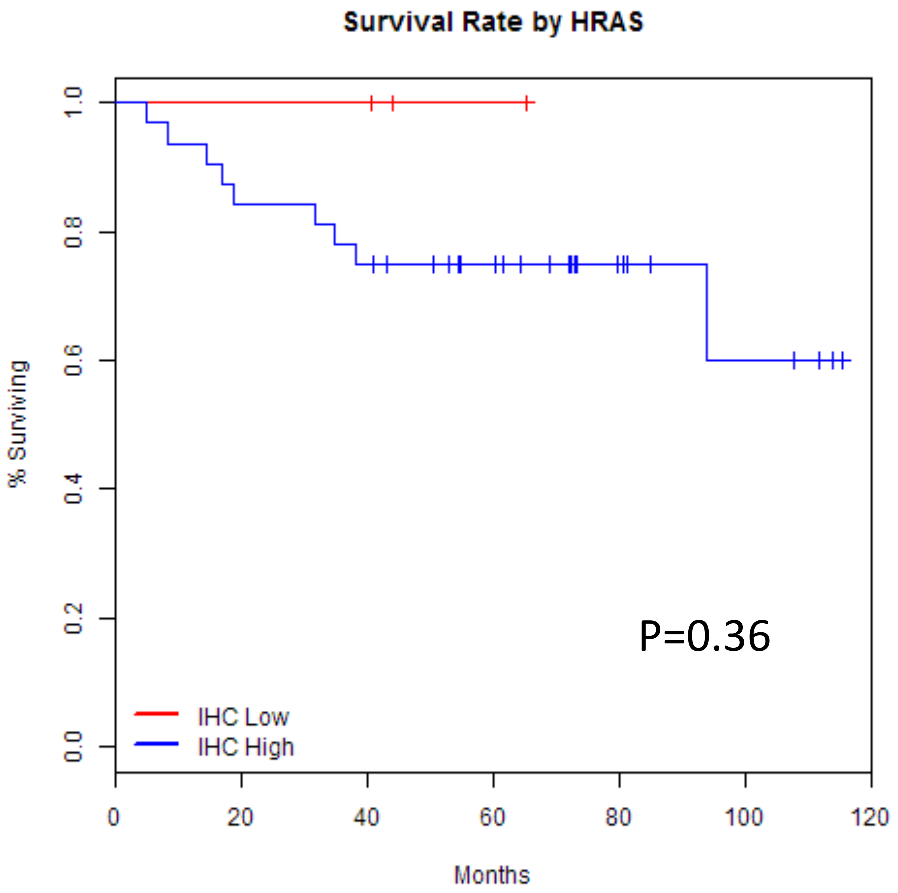

Supplemental Figure 8. Survival curve of IHC expression predicting over all survival of HRAS. Although not significant, the pattern shows better s urvival in IHC low patients.

Supplemental Table 1. Univariate analysis of overall survival (OS) in surgically-treated OSCC patients

Variable	No.	5-year OS (%)	p-value*
Age, <60 vs. ≥60	24/34	81.6/67.9	0.231
Sex, male vs. female	50/8	73.2/100	0.277
T stage, T1-2 vs. T3-4	40/18	77.3/72.5	0.733
N stage, N0-1 vs. N2-3	29/29	73.5/62.1	0.467
Overall stage, I, II vs. III, IV Treatment,	26/32	78.3/62.2	0.439
Tumor margin, clear vs. close/positive	49/9	79.6/72.2	0.526
Extranodal spread, none vs. present	28/30	78.1/69.7	0.578
Treatment, surgery vs. surgery+radiotherapy	16/42	81.0/52.3	0.082
Surgery, transoral vs. transmandibular+transpharyngeal	42/16	73.3/68.7	0.254

*log-rank test

Supplemental Table 2. Copy number variations predicting overall survival most significantly, seen with Cox proportional hazard model, in total patients, including p16+ and p16- oropharyngeal squamous cell carcinomas (n=58). Pattern specifies copy number change from better to worse prognosis.

Cytoband	Genomic Location	Genes	N	Pattern	HR (95% CI)	p-value	chi	p-value	HR(naive)
5q35.1	chr5:170,719,193-170,866,310	**FGF18**, RANBP17, LOC728145, TLX3, RPL19P10, RPL10P8, NPM1	58	= | -	0.25 (0.11-0.56)	0.001	13.07	0.00	-1.40
17p12	chr17:11,355,157-11,435,072	FLJ45455, LOC100289291	58	= | -	0.25 (0.11-0.60)	0.002	11.31	0.00	-1.37
16q24.3	chr16:89,710,830-89,787,100	**CDK10**, CHMP1A, C16orf55, SPATA2L, C16orf7, LOC100128881	58	+ | =	0.24 (0.088-0.64)	0.004	9.55	0.00	-1.44
20p12.1	chr20:17,413,234-17,525,469	**PCSK2**, TCTE1P, BFSP1, RPS27AP2	58	- | = | +	3.17 (1.46-6.87)	0.003	8.24	0.00	1.15
3p25.3	chr3:8,938,984-9,052,691	**RAD18**, SRGAP3	57	+ | = | -	0.31 (0.14-0.73)	0.007	8.04	0.00	-1.16
2q14.3	chr2:123,906,223-124,016,670		57	- | = | +	3.72 (1.47-9.41)	0.006	8.02	0.00	1.31
7p11.2	chr7:54,116,830-54,209,606		57	- | = | +	7.54 (2.09-27.28)	0.002	7.90	0.00	2.02
4p15.32	chr4:17,223,279-17,348,887		58	- | =	3.05 (1.34-6.97)	0.008	7.71	0.01	1.12
7q31.2	chr7:115,818,876-115,905,834	**TES**	57	- | = | +	3.00 (1.34-6.71)	0.007	7.62	0.01	1.10
4q21.21	chr4:81,192,853-81,278,366	**FGF5**, MRPS25P1, C4orf22, LOC100132983	54	- | = | +	4.27 (1.40-13.01)	0.011	7.51	0.01	1.45
1q25.3	chr1:185,261,014-185,345,148	IVNS1ABP, LOC100288079	58	- | = | +	2.55 (1.27-5.12)	0.008	7.24	0.01	0.94
13q13.2	chr13:34,683,844-34,772,336		58	= | -	0.36 (0.16-0.78)	0.010	7.23	0.01	-1.03
11q23.3	chr11:120,203,799-120,287,199	ARHGEF12	58	- | = | +	3.39 (1.35-8.49)	0.009	7.04	0.01	1.22
20p12.2	chr20:10,459,148-10,554,536	C20orf94	58	- | = | +	2.72 (1.27-5.84)	0.010	7.04	0.01	1.00
15q26.3	chr15:102,237,371-102,312,224	TARSL2, LOC100128108, LOC100288426, LOC441734, LOC100289638, LOC100289668, LOC100286891, DNM1P47, LOC441736	57	+ | = | -	0.28 (0.11-0.77)	0.013	6.95	0.01	-1.26
19p13.2	chr19:12,938,345-13,043,682	RTBDN, MAST1, LOC100287872, DNASE2, KLF1, GCDH, RPS6P25, SYCE2, FARSA	58	+ | =	0.30 (0.12-0.77)	0.012	6.94	0.01	-1.19
19p13.3	chr19:2,606,058-2,700,076	GNG7	58	+ | =	0.21 (0.058-0.73)	0.015	6.93	0.01	-1.58
7p14.1	chr7:42,021,742-42,187,019	**GLI3**, LOC100131183	57	- | = | +	6.22 (1.65-23.52)	0.007	6.92	0.01	1.83
20p12.1	chr20:16,655,564-16,787,185	RPL7AP13, SNRPB2, OTOR	58	- | = | +	2.77 (1.28-6.00)	0.010	6.82	0.01	1.02
13q34	chr13:113,986,985-114,060,889	GRTP1, LOC100289508, DKFZp451A211	58	+ | =	0.31 (0.12-0.78)	0.013	6.78	0.01	-1.19
10q26.11	chr10:121,259,097-121,353,089	RGS10, TIAL1	58	+ | = | -	0.24 (0.081-0.71)	0.010	6.77	0.01	-1.43
22q12.1	chr22:28,090,520-28,179,757	MN1	56	- | = | +	4.02 (1.45-11.18)	0.008	6.73	0.01	1.39
19p13.11	chr19:18,639,925-18,709,590	FKBP8, LOC100288150, C19orf50, UBA52, C19orf60, CRLF1	58	+ | =	0.34 (0.14-0.80)	0.014	6.65	0.01	-1.09
9q21.31	chr9:81,605,200-81,705,297	KRT18P24	58	- | = | +	3.10 (1.28-7.55)	0.012	6.31	0.01	1.13
12q21.2	chr12:78,469,689-78,573,894	NAV3	57	- | = | +	2.97 (1.29-6.82)	0.010	6.25	0.01	1.09
8p21.3	chr8:23,110,230-23,187,845	CHMP7, R3HCC1, LOXL2	55	+ | = | -	0.18 (0.04-0.79)	0.024	6.21	0.01	-1.73
7p22.1-7p21.3	chr7:7,264,816-7,353,937	C1GALT1	58	- | = | +	2.49 (1.21-5.14)	0.013	6.21	0.01	0.91
13q12.2	chr13:28,484,673-28,582,916	PDX1, ATP5EP2, LOC100132234, LOC100287444, CDX2, PRHOXNB, FLT3	58	+ | =	0.30 (0.11-0.82)	0.019	6.12	0.01	-1.20
2q31.2	chr2:179,881,263-179,969,516	SESTD1	54	- | = | +	2.52 (1.20-5.27)	0.014	6.11	0.01	0.92
4q35.1	chr4:186,890,697-186,999,717	TLR3	56	- | = | +	3.38 (1.22-9.35)	0.019	6.09	0.01	1.22
20p12.2	chr20:10,792,711-10,870,775	FAT1P1	57	- | = | +	2.54 (1.20-5.36)	0.015	6.09	0.01	0.93
17p11.2	chr17:18,001,993-18,154,255	DRG2, MYO15A, ALKBH5, LLGL1, FLII	58	+ | =	0.28 (0.097-0.83)	0.021	6.06	0.01	-1.26
7p21.3	chr7:9,012,385-9,158,360		58	- | = | +	2.66 (1.19-5.94)	0.017	6.05	0.01	0.98
12p13.33	chr12:410,483-510,446	KDM5A, CCDC77	57	- | = | +	2.40 (1.18-4.88)	0.016	6.03	0.01	0.87
13q34	chr13:114,935,248-115,023,250	LOC100286952, CDC16	58	+ | =	0.35 (0.15-0.84)	0.019	6.00	0.01	-1.04
3p26.3	chr3:500,608-583,065		58	+ | = | -	0.36 (0.16-0.81)	0.014	5.87	0.02	-1.02
5q33.3	chr5:158,840,484-158,960,505	LOC285627	58	= | -	0.37 (0.16-0.86)	0.020	5.82	0.02	-0.99
6q15	chr6:92,448,930-92,529,242	LOC100129847	53	- | = | +	3.06 (1.21-7.73)	0.018	5.79	0.02	1.12
16p13.3	chr16:1,810,188-1,891,929	MAPK8IP3, NME3, MRPS34, EME2, SPSB3, NUBP2, IGFALS, HAGH, FAHD1, C16orf73	55	+ | = | -	0.39 (0.17-0.86)	0.019	5.78	0.02	-0.95
8p23.3	chr8:260,715-362,664	FAM87A, LOC100288862, FBXO25	58	+ | = | -	0.43 (0.21-0.87)	0.018	5.72	0.02	-0.85
8q12.1	chr8:57,002,533-57,074,440	NPM1P21, MOS, PLAG1	58	+ | =	0.25 (0.075-0.85)	0.027	5.69	0.02	-1.37
6q23.3	chr6:138,081,361-138,186,385		58	- | =	4.86 (1.15-20.57)	0.032	5.63	0.02	1.58
1p34.3	chr1:36,192,287-36,296,144	LOC100289612, CLSPN, EIF2C4	58	+ | = | -	0.34 (0.14-0.81)	0.015	5.61	0.02	-1.07
20p12.1	chr20:13,686,151-13,821,855	ESF1, C20orf7	57	- | = | +	2.80 (1.19-6.56)	0.018	5.36	0.02	1.03
11q21	chr11:95,078,661-95,253,377		58	- | = | +	2.96 (1.14-7.66)	0.025	5.34	0.02	1.09
18p11.32	chr18:1,842,435-1,947,071	LOC100128360	58	- | = | +	2.66 (1.17-6.02)	0.019	5.23	0.02	0.98
7q21.11	chr7:80,236,665-80,322,097	CD36	58	- | = | +	2.44 (1.12-5.35)	0.025	5.23	0.02	0.89
2q14.1	chr2:118,424,947-118,539,527		58	- | =	2.49 (1.11-5.62)	0.027	5.21	0.02	0.91
11q23.3	chr11:119,212,271-119,295,210	MFRP, C1QTNF5, MFRP-C1QTNF5, USP2, LOC100130353, THY1	58	+ | = | -	0.46 (0.24-0.91)	0.024	5.17	0.02	-0.77
2q23.3	chr2:152,330,458-152,467,656	RIF1, NEB	57	- | = | +	3.31 (1.11-9.86)	0.031	5.14	0.02	1.20
20q11.21	chr20:30,738,720-30,827,765	TM9SF4, RPL24P1, TSPYL3, PLAGL2, POFUT1, MIR1825	58	+ | =	0.41 (0.18-0.91)	0.028	5.13	0.02	-0.89
12p13.1	chr12:13,399,154-13,469,241		58	- | = | +	2.59 (1.14-5.89)	0.023	5.12	0.02	0.95
3p26.2	chr3:3,302,261-3,421,487		58	= | -	0.42 (0.20-0.92)	0.029	5.06	0.02	-0.86
4q28.1	chr4:125,987,550-126,069,926		55	- | = | +	2.59 (1.15-5.84)	0.022	5.05	0.02	0.95
7p15.3	chr7:21,270,389-21,385,748		57	- | = | +	4.12 (1.18-14.33)	0.026	5.04	0.02	1.42

+, gain; =, normal (neutral); -, loss.

Supplemental Table 3. Copy number variations predicting overall survival most significantly, seen with Cox proportional hazard model, in p16+ oropharyngeal squamous cell carcinomas (n=35). Pattern specifies copy number change from better to worse prognosis.

Cytoband	Genomic Location	Genes	N	Pattern	HR (95% CI)	p-value	chi	p-value	HR(naive)
11p15.5	chr11:509,204-661,925	HRAS, LOC100128703, LRRC56, C11orf35, RASSF7, MIR210, LOC100288751, LOC143666, PHRF1, IRF7, MUPCDH, SCT, DRD4, DEAF1	35	+ | =	0.10 (0.012-0.82)	0.03	7.02	0.01	-2.30
6q25.3	chr6:160,102,668-160,204,138	SOD2, LOC100132803, WTAP, LOC100129518, ACAT2, TCP1, SNORA20	34	+ | = | -	0.00 (0.00-Inf)	1.00	6.89	0.01	-21.07
2p12	chr2:79,287,399-79,398,378	REG1B, REG1A, REG1P, REG3A	35	- | =	5.32 (1.32-21.45)	0.02	6.87	0.01	1.67
8p23.1	chr8:10,914,839-11,025,183	XKR6	35	= | -	0.16 (0.032-0.78)	0.02	6.68	0.01	-1.84
4q12	chr4:55,832,085-55,988,295	KDR, RPL38P3	35	- | =	9.18 (1.13-74.78)	0.04	6.35	0.01	2.22
5p15.31	chr5:8,306,218-8,403,777		35	- | = | +	3.89 (1.25-12.10)	0.02	6.30	0.01	1.36
3q26.1	chr3:162,425,550-162,629,217		33	- | = | +	9.36 (1.13-77.67)	0.04	6.23	0.01	2.24
2q14.1	chr2:118,424,947-118,539,527		35	- | =	5.99 (1.22-29.42)	0.03	6.18	0.01	1.79
7p22.1-7p21.3	chr7:7,264,816-7,353,937	C1GALT1	35	- | = | +	4.86 (1.37-17.21)	0.01	6.11	0.01	1.58
11p13	chr11:35,826,201-35,910,591	TRIM44, KRT18P14	31	+ | = | -	0.21 (0.051-0.83)	0.03	6.00	0.01	-1.58
2q37.2	chr2:235,633,702-235,719,621		35	+ | = | -	0.20 (0.048-0.80)	0.02	5.93	0.01	-1.62
13q21.2	chr13:59,909,909-60,037,612		35	- | =	5.80 (1.17-28.82)	0.03	5.91	0.02	1.76
19p13.11	chr19:18,639,925-18,709,590	FKBP8, LOC100288150, C19orf50, UBA52, C19orf60, CRLF1	35	+ | =	0.17 (0.035-0.86)	0.03	5.80	0.02	-1.75
4q22.1	chr4:88,986,536-89,140,493	PKD2, ABCG2, RPL31P24	35	- | =	8.06 (0.99-65.61)	0.05	5.39	0.02	2.09
20q11.21	chr20:30,738,720-30,827,765	TM9SF4, RPL24P1, TSPYL3, PLAGL2, POFUT1, MIR1825	35	+ | =	0.19 (0.038-0.92)	0.04	5.31	0.02	-1.68
20p12.1	chr20:17,413,234-17,525,469	PCSK2, TCTE1P, BFSP1, RPS27AP2	35	- | = | +	4.56 (1.27-16.39)	0.02	5.28	0.02	1.52
6p21.33	chr6:31,509,719-31,594,903	BAT1, ATP6V1G2, NFKBIL1, LOC100289233, LOC100287329, LTA, TNF, LTB, LST1, NCR3, LOC100130756, AIF1, BAT2, SNORA38	35	+ | =	0.13 (0.016-1.06)	0.06	5.06	0.02	-2.04
6q23.3	chr6:138,081,361-138,186,385		35	- | =	373578717 (0.00-Inf)	1.00	5.00	0.03	19.74
11q23.3	chr11:120,203,799-120,287,199	ARHGEF12	35	- | =	402463477 (0.00-Inf)	1.00	4.98	0.03	19.81
21q22.3	chr21:46,271,616-46,366,018	PTTG1IP, ITGB2, C21orf67, C21orf70	35	+ | = | -	0.32 (0.11-0.91)	0.03	4.85	0.03	-1.15
19p13.2	chr19:11,435,403-11,562,430	TSPAN16, RAB3D, TMEM205, LOC126075, LPPR2, C19orf39, EPOR, RGL3, CCDC151, PRKCSH, ELAVL3	35	+ | =	0.14 (0.018-1.14)	0.07	4.56	0.03	-1.96
4q13.3	chr4:74,761,205-74,920,746	LOC642958, CXCL1P, PF4, PPBP, CXCL5, CXCL3, PPBPL2	35	- | =	7.08 (0.87-57.65)	0.07	4.56	0.03	1.96
16q23.3	chr16:83,650,499-83,752,836	CDH13	35	- | =	4.07 (1.00-16.46)	0.05	4.51	0.03	1.40
4q31.22	chr4:147,523,515-147,659,972	POU4F2, TTC29	35	- | =	4.80 (0.97-23.90)	0.06	4.40	0.04	1.57
19q13.33	chr19:49,389,685-49,469,887	TULP2, NUCB1, DHDH, BAX, FTL	35	+ | =	0.21 (0.042-1.04)	0.06	4.40	0.04	-1.57
3p21.2	chr3:51,944,835-52,027,083	RRP9, PARP3, GPR62, PCBP4, LOC100287670, ABHD14B, LOC100289334, ABHD14A, ACY1	35	+ | =	0.00 (0.00-Inf)	1.00	4.34	0.04	-19.72
20q11.21	chr20:30,102,225-30,124,886	HM13	35	+ | =	0.25 (0.063-1.02)	0.05	4.33	0.04	-1.37
2q35	chr2:219,119,542-219,188,809	GPBAR1, AAMP, PNKD, LOC100288953, TMBIM1	35	+ | =	0.21 (0.043-1.06)	0.06	4.33	0.04	-1.54
4q28.3	chr4:134,415,105-134,507,251		33	- | = | +	7.20 (0.86-60.31)	0.07	4.33	0.04	1.97
4q21.21	chr4:81,192,853-81,278,366	FGF5, MRPS25P1, C4orf22, LOC100132983	32	- | = | +	399962088 (0.00-Inf)	1.00	4.32	0.04	19.81
16q21	chr16:65,208,720-65,339,533	LOC283867	35	- | =	3.92 (0.98-15.72)	0.05	4.32	0.04	1.37
15q26.3	chr15:102,237,371-102,312,224	TARSL2, LOC100128108, LOC100288426, LOC441734, LOC100289638, LOC100289668, LOC100286891, DNM1P47, LOC441736	35	+ | =	0.15 (0.018-1.21)	0.07	4.27	0.04	-1.91
19p13.2	chr19:12,938,345-13,043,682	RTBDN, MAST1, LOC100287872, DNASE2, KLF1, GCDH, RPS6P25, SYCE2, FARSA	35	+ | =	0.15 (0.018-1.21)	0.07	4.27	0.04	-1.91
4q31.1	chr4:140,708,794-140,826,227	MAML3	34	- | = | +	7.44 (0.86-64.43)	0.07	4.26	0.04	2.01
6q15	chr6:92,448,930-92,529,242	LOC100129847	32	- | = | +	433088796 (0.00-Inf)	1.00	4.15	0.04	19.89
12q24.33	chr12:132,214,338-132,296,104	SFRS8	35	- | = | +	3.24 (1.02-10.26)	0.05	4.14	0.04	1.18
2q22.1	chr2:139,848,343-139,985,657		35	- | =	4.61 (0.92-23.00)	0.06	4.11	0.04	1.53
2q35	chr2:220,399,553-220,533,109	ACCN4, CHPF, TMEM198, OBSL1, INHA, STK11IP, SLC4A3	35	+ | = | -	0.31 (0.098-1.00)	0.05	4.05	0.04	-1.16
10q23.1	chr10:83,264,655-83,357,595		34	- | = | +	351949282 (0.00-Inf)	1.00	4.02	0.04	19.68
4q31.21	chr4:145,173,445-145,282,750		35	- | =	7.15 (0.82-62.35)	0.07	4.01	0.05	1.97
8q12.1	chr8:57,002,533-57,074,440	NPM1P21, MOS, PLAG1	35	+ | =	0.14 (0.016-1.22)	0.07	4.01	0.05	-1.97
8q23.3	chr8:116,672,709-116,763,331	TRPS1	35	= | +	4.54 (0.91-22.64)	0.07	4.01	0.05	1.51
20q11.21	chr20:31,351,378-31,432,790	DNMT3B, MAPRE1	35	+ | =	0.16 (0.02-1.26)	0.08	4.01	0.05	-1.85
6q27	chr6:168,311,394-168,395,997	MLLT4, HGC6.3	35	+ | =	0.23 (0.047-1.11)	0.07	3.97	0.05	-1.47

+, gain; =, normal (neutral); -, loss.

Supplemental Table 4. Survival rate by copy number alteration of oncogene reported to be highly amplified in HNSCC: EGFR, CCND1, MYC, FGFR1, and PIK3CA, seen in groups of all samples, HPV p16+, and p16-. None of them showed statistical significance to predict survival in HNSCC.

Oncogene	Subset	n	HR	HR (95% CI)	p-value	X2	p-value	HR(naive)
	all	58	1.30	(0.62-2.74)	0.48	0.49	0.48	0.27
EGFR	p16+	35	0.84	(0.22-3.20)	0.80	0.06	0.80	-0.17
	p16-	23	0.91	(0.35-2.38)	0.86	0.03	0.86	-0.09
	all	58	1.64	(0.68-3.94)	0.27	1.25	0.26	0.49
CCND1	p16+	35	2.16	(0.47- 9.97)	0.32	1.02	0.31	0.77
	p16-	23	0.61	(0.20-1.92)	0.40	0.72	0.40	-0.49
	all	58	1.02	(0.51-2.02)	0.95	0.00	0.95	0.02
MYC	p16+	35	0.96	(0.33-2.84)	0.95	0.00	0.95	-0.04
	p16-	23	1.31	(0.50-3.41)	0.59	0.30	0.58	0.27
	all	58	1.03	(0.39-2.73)	0.95	0.00	0.95	0.03
FGFR1	p16+	35	2.18	(0.54-8.76)	0.27	1.28	0.26	0.78
	p16-	23	0.53	(0.12-2.31)	0.39	0.75	0.39	-0.64
	all	58	1.10	(0.66-1.85)	0.71	0.14	0.71	0.10
PIK3CA	p16+	35	1.26	(0.53-3.01)	0.60	0.27	0.60	0.23
	p16-	23	1.07	(0.51-2.24)	0.86	0.03	0.86	0.07
